# Effects of resveratrol on cariogenic virulence properties of *Streptococcus mutans*

**DOI:** 10.1186/s12866-020-01761-3

**Published:** 2020-04-17

**Authors:** Jinheng Li, Tiantian Wu, Weiwei Peng, Yaqin Zhu

**Affiliations:** Department of General Dentistry, College of Stomatology, Shanghai Ninth People’s Hospital, Shanghai Jiao Tong University School of Medicine, National Clinical Research Center for Oral Diseases, Shanghai Key Laboratory of Stomatology & Shanghai Research Institute of Stomatology, 639 Zhi Zao Ju Road, Shanghai, 200011 China

**Keywords:** Resveratrol, *Streptococcus mutans*, Acidogenicity, Aciduricity, Extracellular polysaccharide, Biofilm

## Abstract

**Background:**

*Streptococcus mutans* is the principal etiological agent of human dental caries. The major virulence factors of *S. mutans* are acid production, acid tolerance, extracellular polysaccharide (EPS) synthesis and biofilm formation. The aim of this study is to evaluate the effect of resveratrol, a natural compound, on virulence properties of *S. mutans*.

**Results:**

Resveratrol at sub-MIC levels significantly decreased acid production and acid tolerance, inhibited synthesis of water-soluble polysaccharide and water-insoluble polysaccharide, compromised biofilm formation. Related virulence gene expression (*ldh, relA, gtfC, comDE*) was down-regulated with increasing concentrations of resveratrol.

**Conclusions:**

Resveratrol has an inhibitory effect on *S. mutans* cariogenic virulence properties and it represents a promising anticariogenic agent.

## Background

Dental caries is a multi-factorial infectious chronic disease which causes demineralization and progressive destruction of the dental enamel by specific bacteria and their virulence products [1]. *Streptococcus mutans*, a Gram-positive oral bacterium, has long been implicated as the principal etiological agent of human dental caries [2]. The major virulence factors of *S. mutans* are its ability to produce organic acids through metabolism of dietary carbohydrates (acidogenecity) and to withstand and survive under low pH environment (aciduricity) [3, 4]. One of the most important virulence factors is its ability to produce glucosyltransferases (GTFs) to catalyze synthesis of extracellular polysaccharides (EPS) from sucrose, which allows bacteria to effectively colonize on the tooth surfaces and contribute to the formation of highly cariogenic plaque biofilms [5, 6]. Therefore, inhibition of cariogenic virulence of *S. mutans* could be an effective way to prevent and control dental caries.

Fluoride plays an important role in preventing the prevalence and severity of dental caries [7]. It is a well-known cariostatic agent via the inhibition of demineralization and the enhancement of remineralization and the inhibition of bacterial activity such as acid production, acid tolerance and EPS formation [8]. However, its excessive use results in adverse effects like fluorosis which limited fluoride using for public health in many countries [9, 10]. Therefore, the development of an alternative cariostatic agent with minimal side effects is urgent.

Natural products are major sources of attractive and effective therapeutic agents for the discovery and development of new drugs throughout human history. They have a wide range of structurally biochemical specificities and can divided into phenolic acids, anthraquinones, flavonoids, stilbenes, tannins, terpenoids and alkaloids [11, 12]. Recently, there is an increasing interest in natural products as cariostatic agents for promising novel anti-cariogenic strategy. A number of compounds, such as epicatechin, apigenin, oolong tea, have shown their efficacy anti-caries activity against oral microbial pathogens *S. mutans* [13–15]*.*

Resveratrol (3,5,4′-trihydroxy-*trans*-stilbene) is a natural compound found in many plant extracts, including grapes, peanuts, cranberries and *Polygonum cuspidatums*. It is a member of the stilbene family and a precursor of other stilbenes such as viniferins and pterostilbene (trans-3,5-dimethoxy-40-hydroxystilbene). Resveratrol is known to have numerous biological functions such as antimicrobial activity, antiviral, antioxidant, anti-inflammatory, and anticancer [16, 17]. But there is little work about its anti-cariogenic properties.

Therefore, in the present study, we investigated the effect of resveratrol on *S. mutans* anti-cariogenic properties. We focused on the effects of resveratrol on acid production, acid tolerance, extracellular polysaccharide synthesis, biofilm formation and structure, virulence gene expression. This research would help to offer the possibility of natural product as novel anti-cariogenic agent to advance the caries progression investigation and prevent dental diseases efficiently.

## Results

### Growth curve assay and MIC

**Fig. 1:**
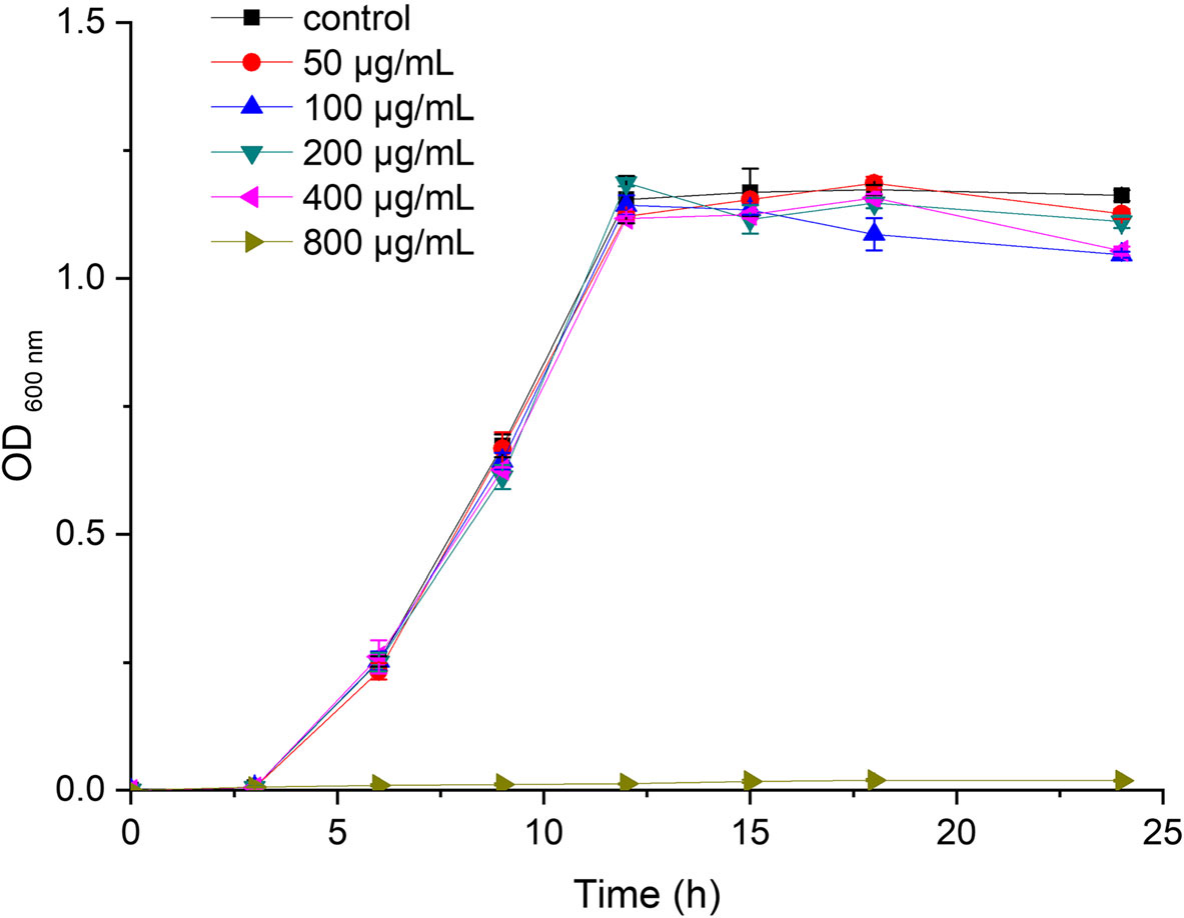
Growth curve of *S.mutans* with different concentrations of resveratrol

We evaluated the effects of resveratrol with different concentrations on growth rate of *S. mutans* by growth curve assay. It was observed that in comparison to the vehicle control, the bacterial growth was significantly inhibited with 800 μg/mL resveratrol treatment (Fig. 1). The MIC of resveratrol against *S. mutans* was 800 μg/mL. However, there was no obvious difference in the growth curve with resveratrol concentrations below 400 μg/mL.

### Acid production assay

**Fig. 2:**
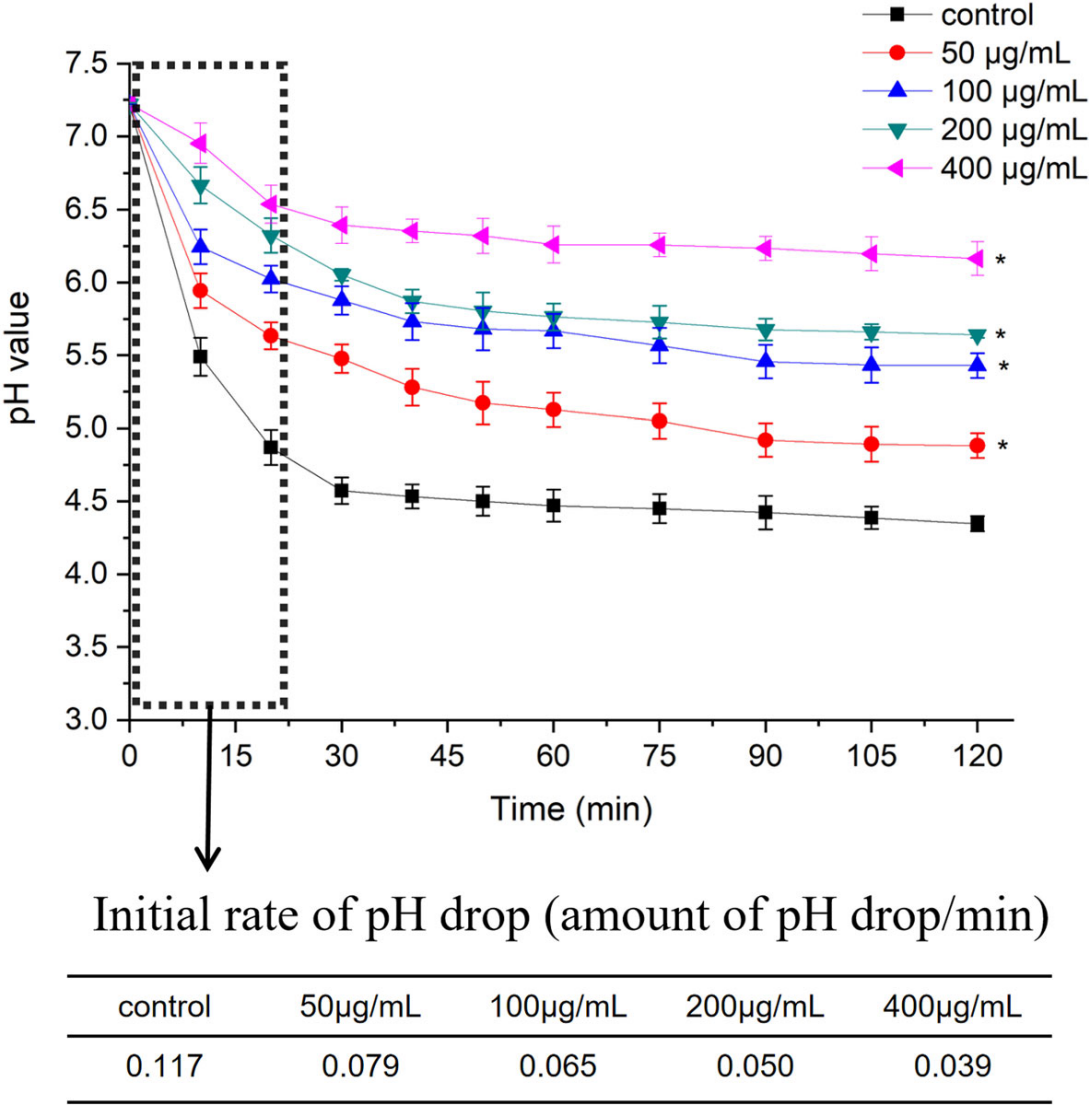
Effect of resveratrol on Glycolytic pH drop. *Statistically significant differences (*P* < 0.05) between with or without resveratrol

**Fig. 3:**
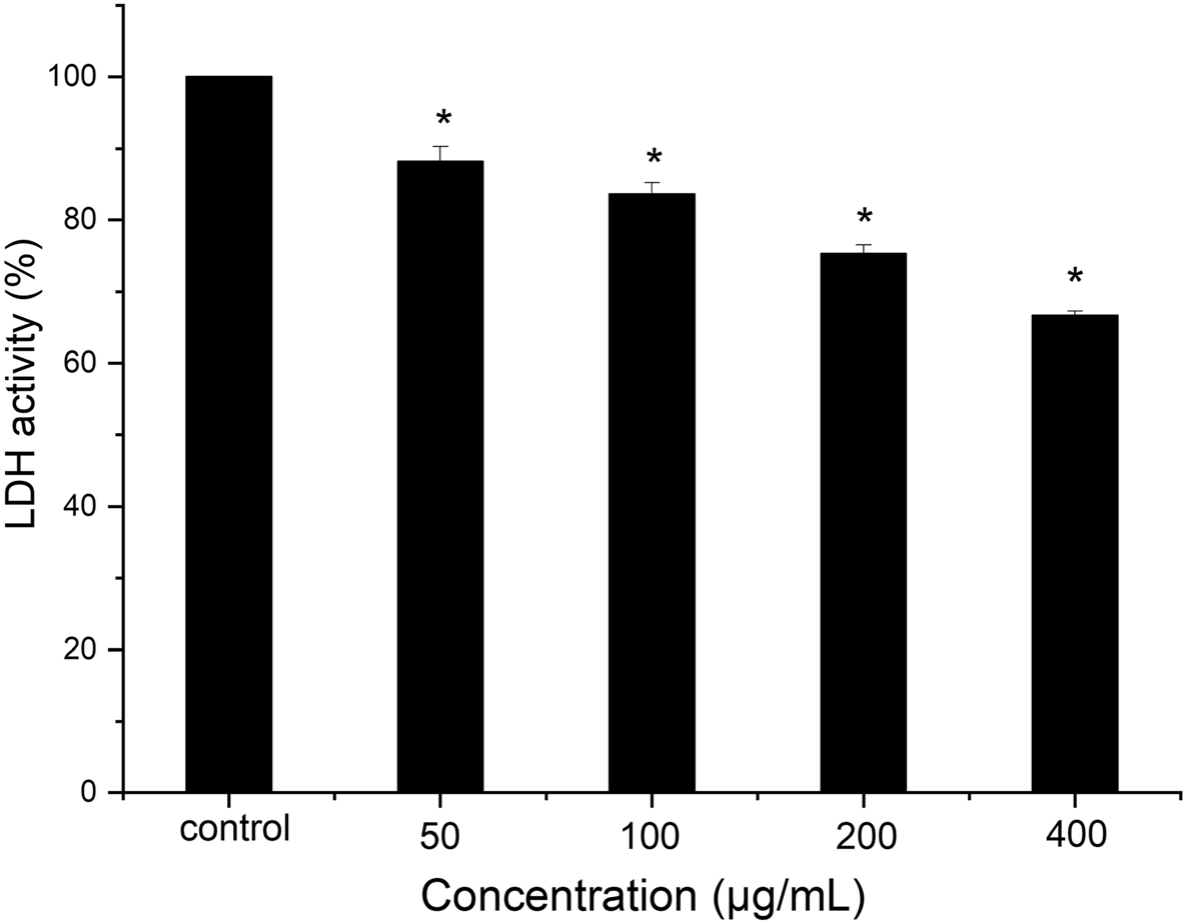
Effect of resveratrol LDH activity. *Statistically significant differences (*P* < 0.05) between with or without resveratrol

We determined the effects of resveratrol at sub-MIC levels on acid production through the glycolytic pH drop assay and LDH assay. As shown in Fig. 2, the pH value (at 10, 20, 30, 40, 50, 60, 75, 90, 105 and 120 min incubation period) at 50, 100, 200 and 400 μg/mL resveratrol were significantly different from those of the vehicle control (*P* < 0.05). In general, resveratrol reduced the initial rate of the pH drop (the amount of pH drop/min) at 50, 100, 200 and 400 μg/mL, compared with the vehicle control (*P* < 0.05). The final pH values (at 120 min incubation) at 50, 100, 200 and 400 μg/mL resveratrol were significantly different from that of the vehicle control (P < 0.05). As shown in Fig. 3, LDH activity was decreased from 88.2 ± 2.1% at 50 μg/mL to 66.7 ± 0.6% at 400 μg/mL, resveratrol significantly reduced LHD activity at 50, 100, 200 and 400 μg/mL compared with the control (*P* < 0.05), which were consistent with the results of the glycolytic pH drop assay.

### Acid tolerance assay

**Fig. 4:**
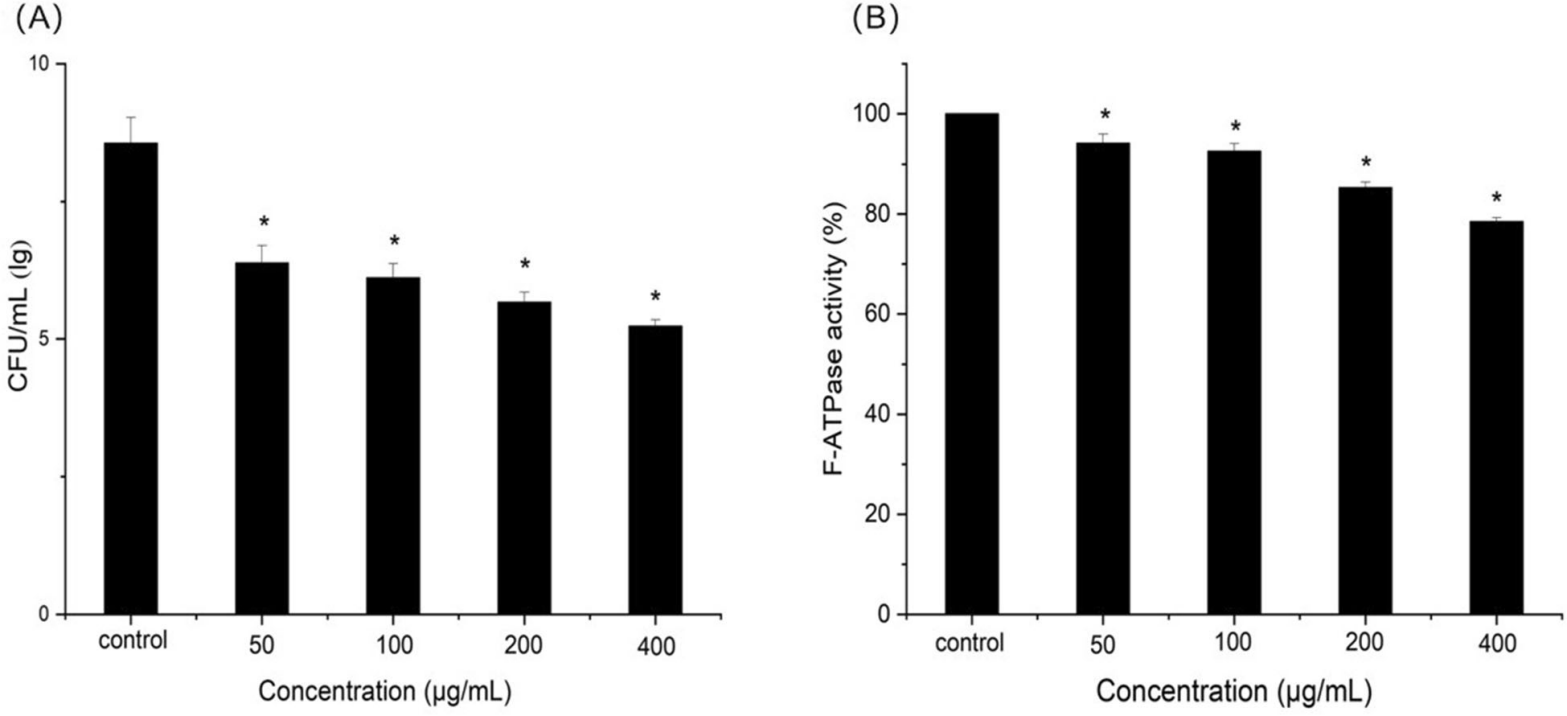
Effect of resveratrol on acid production. **a** Survival rate of S. mutans at pH 5.0, **b** F-ATPase activity*.* *Statistically significant differences (*P* < 0.05) between with or without resveratrol

Resveratrol at sub-MIC levels can also inhibit *S. mutans* acid tolerance. As shown in Fig. 4a, The survive rate of *S. mutans* at pH 5.0 was significantly decreased after treatment with resveratrol at 50, 100, 200 and 400 μg/mL compared with the control (*P* < 0.05). At the same time, F-ATPase activity assay was performed to confirm the effect of resveratrol on acid tolerance. The F-ATPase activity of *S. mutans* was decreased from 94.2 ± 1.8% at 50 μg/mL to 78.5 ± 0.8% at 400 μg/mL compared with the control as shown in Fig. 4b. This indicated that resveratrol had a significant inhibitory effect on F-ATPase compared with the control (*P* < 0.05).

### Polysaccharide analyses

**Fig. 5:**
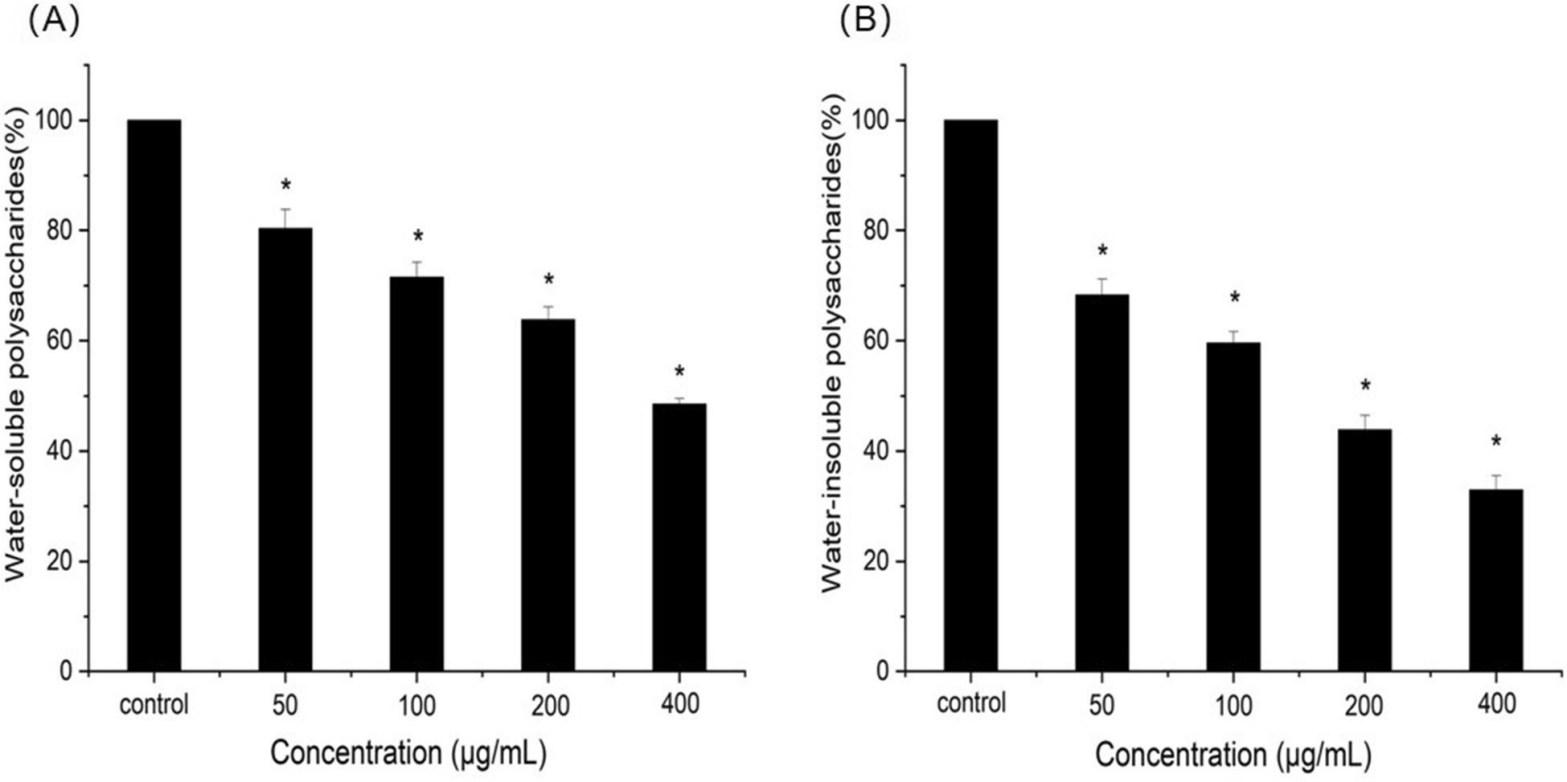
Effect of resveratrol on extracellular polysaccharide. **a** Water-soluble polysaccharide, **b** Water-insoluble polysaccharide. *Statistically significant differences (*P* < 0.05) between with or without resveratrol

Polysaccharide including water-soluble polysaccharide and water-insoluble polysaccharide was measured by phenol-sulfuric acid method. The results were shown in Fig. 5a and b. Compared with the control group, the water-soluble polysaccharide produced by *S. mutans* was reduced by 20–50% at different concentration of resveratrol, while the water-insoluble polysaccharide was reduced by 30–70%. Resveratrol can significantly inhibit production of water-.

Soluble and water-insoluble polysaccharides compared with the control (*P* < 0.05).

### Biofilm biomass

**Fig. 6:**
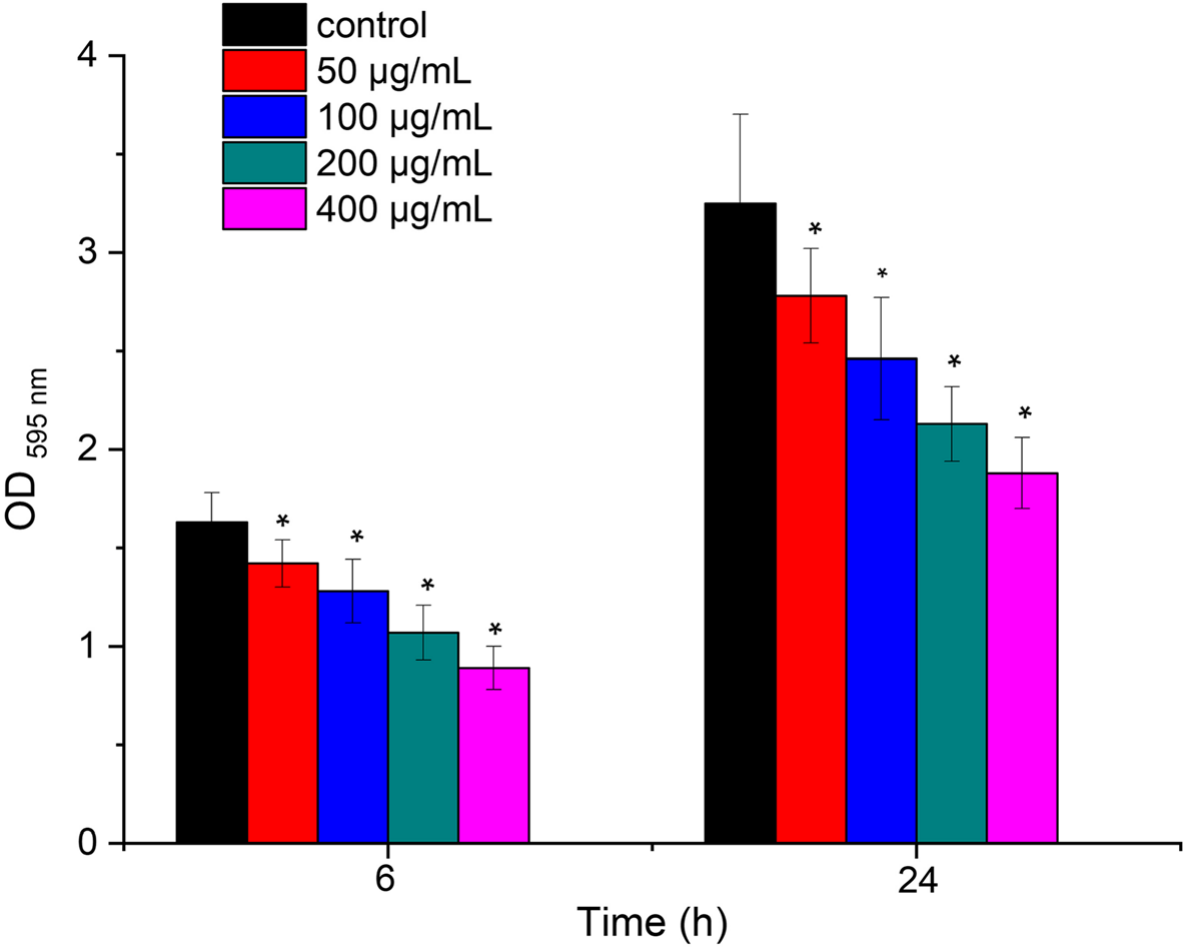
Effect of resveratrol on biofilm formation at 6 h and 24 h time points. *Statistically significant differences (*P* < 0.05) between with or without resveratrol at 6 h and 24 h time points

The quantification of biofilm formed by *S. mutans* with concentrations of resveratrol (0, 50, 100, 200, 400 μg/mL) were shown in Fig. 6. There were significant differences in the overall biomass of biofilms at both 6 h and 24 h time points for different concentrations of resveratrol compared with the control (P < 0.05). After 6 h incubation, the OD _595 nm_ values of the biofilm formed by *S. mutans* was 1.63 ± 0.15. With increasing concentration of resveratrol, the OD _595 nm_ values decreased from 1.42 ± 0.12 at 50 μg/mL to 0.89 ± 0.11 at 400 μg/mL. A similar trend was observed after 24 h. The OD _595 nm_ values decreased from 3.25 ± 0.45 in the absence of resveratrol to 1.88 ± 0.18 in the presence of 400 μg/mL resveratrol. These results indicated that resveratrol can inhibit *S. mutans* biofilm formation at different time points.

### Biofilm structure

**Fig. 7:**
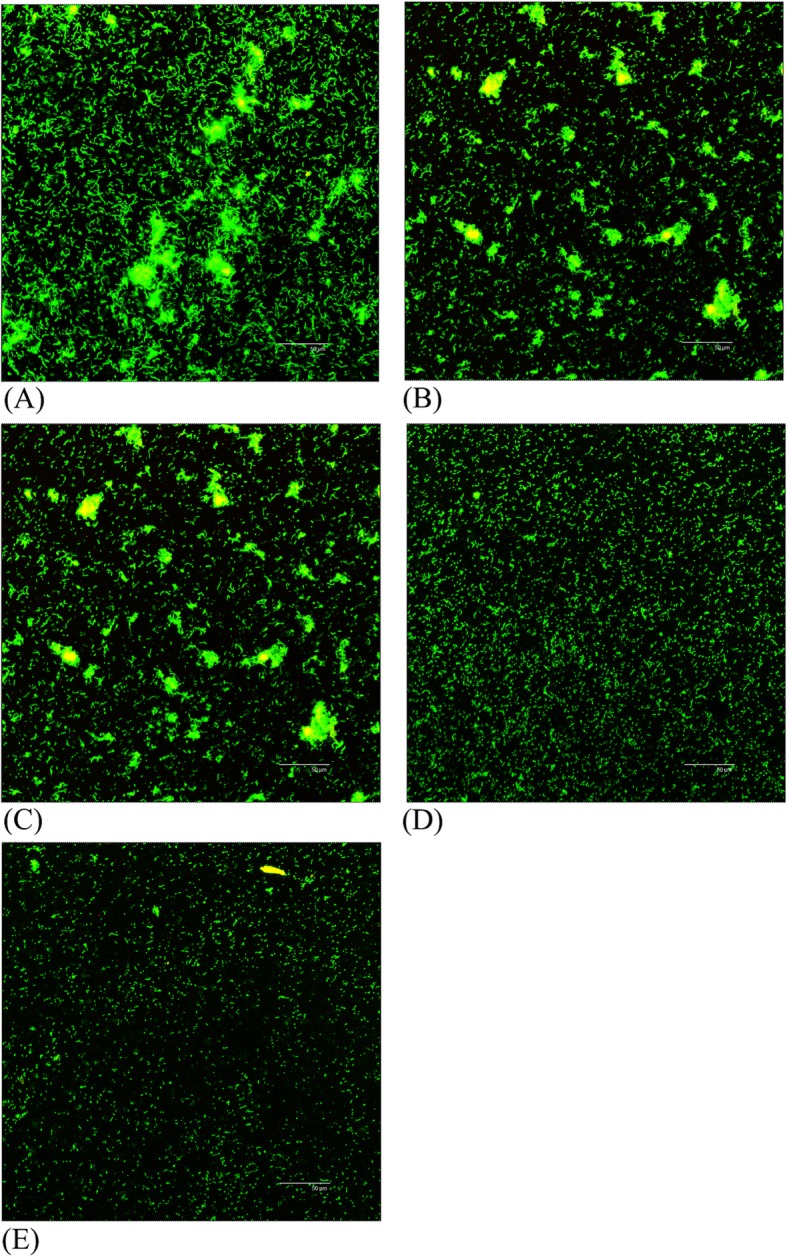
Effect of resveratrol on biofilm structure by confocal laser scanning micrographs. (**a**) 0 μg/mL (**b**) 50 μg/mL (**c**) 100 μg/mL (**d**) 200 μg/mL (**e**) 400 μg/mL. Bar = 50 μm

The structures of the biofilms formed on glass-bottom chamber slides treatment with different concentrations of resveratrol were shown in Fig. 7. The biofilms generated by *S. mutans* without resveratrol aggregated into distinct clusters with a thick, dense structure (Fig. 7a). Following treatment with resveratrol, the biofilms appeared more looser and disperser (Fig. 7b-e). According to the CLSM images, the biofilm thickness after 24 h of cultivation without resveratrol was 26.69 ± 1.78 μm. In the presence of increasing concentration of resveratrol, the biofilm thicknesses became significantly thinner (*P* < 0.05), and it was only approximately 10.37 ± 0.71 μm at a 400 μg/mL concentration of resveratrol.

### Gene expression

**Fig. 8:**
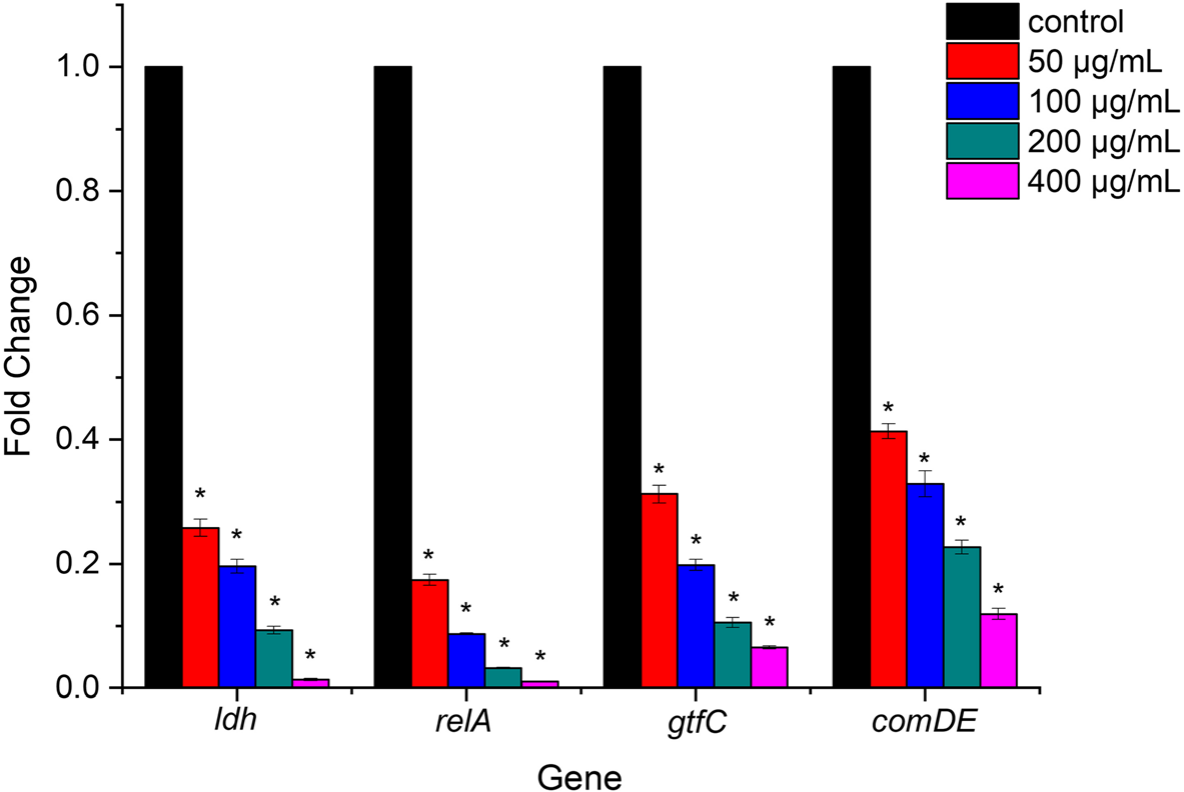
Effect of resveratrol on gene expression of *S.mutans* biofilms. *Statistically significant differences (*P* < 0.05) in gene expression between with or without resveratrol

The expression changes of four genes involved in acid production (*ldh*), acid tolerance (*relA*), extracellular polysaccharide synthesis (*gtfC*) and biofilm formation (*comDE*) in *S. mutans* treated with different concentration of resveratrol were quantified by real time RT-PCR. As shown in Fig. 8, all virulence genes tested was significantly down regulated after resveratrol treatment compared to the control group (*P* < 0.05). Expression levels in tested genes (*ldh*, *relA*, *gtfC*, *comDE*) expression levels treated with 50 μg/mL resveratrol were reduced 0.258, 0.174, 0.312 and 0.413-fold respectively. The down-regulation gene expression was in a dose-dependent manner with resveratrol concentrations. Among them, expression of *relA* were most significantly decreased by 0.0099-fold after 400 μg/mL resveratrol treatment. Expression levels in other genes (*ldh*, *gtfC*, *comDE*) expression levels under 400 μg/mL resveratrol treatment were decreased 0.013, 0.065and 0.119 -fold respectively.

## Discussion

Dental caries is one of the most common oral infectious disease and a major health problem affecting thousands of people worldwide. *S. mutans* is the primary etiological agent of dental caries. Although several antimicrobial agents are used for the prevention of dental caries, the search for an effective agent with minimal side effects is still urgent. Under this background, nature plant products have attracted great attention in recent years. Previous researches studied the effect of the substances separated from *Polygonum cuspidatum* root on the development of dental caries [18–20]. Among them, a fraction (F1) was mainly composed of resveratrol, emodin and physcion (approximately 16.2, 18.9 and 2.07%, respectively). F1, alone or in combination with fluoride, has the ability to disrupt virulence factors of *S. mutans* biofilms. Another fraction F3 which consisted mainly of resveratrol and emodin (approximately 60%) showed the strongest inhibitory effect on the acidogenicity of *S. mutans*. Since these studies were carried out on crude extracts, we investigated the effect of resveratrol on *S. mutans* anti-cariogenic properties in this article.

Cariostatic effect can be obtained through reducing the production of acid by cariogenic bacteria or inhibiting the activity of enzyme associated with the glycolysing systems [21]. Glycolysis is the main pathway to produce acid, and LDH known for lactic acid production is an important enzyme in the process of *S. mutans* [22]. Therefore, this study investigated the acid production through glycolytic acid production assay and LDH activity assay. A reduction in the initial rate of pH drop and LDH activity with resveratrol were shown in Fig. 2. These findings suggested that resveratrol reduced the acid production rates of *S. mutans* at sub-MIC levels due to the suppression of bacterial glycolytic pathway. Moreover, the final pH values in glycolytic pH drop assay with 200 and 400 μg/mL resveratrol were much higher than the critical pH value which balances the rate of demineralization and remineralization of tooth enamel [23]. The final pH values also implied acid tolerance, another key virulence property in *S. mutans* [24]*.* Our results also showed that the number of surviving S. mutans at pH 5.0 was significantly reduced by resveratrol. In order to confirm the inhibitory effects of resveratrol on acid tolerance, we performed proton-permeability and F-ATPase activity assays. F-ATPase maintains pH gradient across the cell membrane, which is related to acid tolerance [25]. The inhibition of F-ATPase enzymatic activity by resveratrol observed in this study may contribute to a rise in the cytoplasmic acidity, followed by decreasing acid adaptation. Thus, it is evidently that inhibitory effect of resveratrol on acid tolerance is attributed, or at least in part, to reduction of F-ATPase activity.

*S. mutans* produce water soluble and insoluble extracellular polysaccharides (EPS) which mediate the adherence and colonization in the oral cavity [26]. Furthermore, insoluble extracellular polysaccharides are particularly important for the formation and structural integrity of dental biofilm matrix [27]. In the present study, resveratrol reduced both water soluble and water insoluble polysaccharides and the reduction of insoluble polysaccharide was more significantly, which will inhibit adherence and biofilm formation. We also investigated the effect of resveratrol on biofilm formation. Biofilm formation is a complex process that begins with the initial adherence to a substrate surface, reversibly and irreversible attachment, and finally matures into integrated biological structure [28, 29]. The results of CV assays showed that resveratrol reduced biofilm formation during different growth phases including initial adherence phase (6 h) and maturation phase (24 h) at sub-MIC level. This is in agreement with CLSM images that the biofilms became much looser and thinner after treatment with resveratrol. These results suggested that resveratrol can efficiently inhibit the biofilm formation not by decreasing in bacterial viability.

Additionally, we also investigated the effect of resveratrol on various cariogenic virulence factors of *S. mutans* at transcriptional levels. The expression profile of virulence genes including acid production, acid tolerance, extracellular polysaccharide synthesis and biofilm formation. Real time PCR results showed that the lactate dehydrogenase (*ldh*) gene expression were down regulated which was consistent with the LDH activity assay. *RelA* gene encodes guanosine tetra (penta)-phosphatesynthetase be involved in the oxidative stress and acid tolerance mechanisms [30]. Our results found that this gene expression was reduced which impaired acid tolerance of *S. mutans*. GTFC (encoded by *gtfC*), catalyse the synthesis of water insoluble and alkali soluble glucan from sucrose with both α-1,3 and α-1,6-linked glucans, which are required for biofilm formation and structurally stable biofilms. The *gtfC* Mutant strains of *S. mutans* is less cariogenic than the wild type strains in vivo [31, 32]. The ComDE system is the most common intraspecific cell-cell communication quorum sensing system in *S. mutans*. The quorum sensing system is an essential component of entire gene regulation networks responsible for the adaptation of bacteria in biofilms [33, 34]. It can respond to environmental fluctuations and mediate a number of physiological virulence activities including biofilm formation. Inactivation of any component of *S. mutans* ComCDE pathway resulted in a phenotype that was biofilm-defective [35]. The repression of this gene would attenuate internal communication quorum sensing mechanism in *S. mutans* and further inhibit biofilm formation. Therefore, the reduction in the expression of tested genes will thereby suppress a series of various cariogenic virulence factors in *S. mutans*.

## Conclusions

Resveratrol, a natural compound found in plant, has an inhibitory effect at sub-MIC level on *S. mutans* cariogenic virulence factors including acid production, acid tolerance, extracellular polysaccharide synthesis, biofilm formation and structure, virulence gene expression. This study has laid the foundation for resveratrol as a new natural product to inhibit the physiological activity of cariogenic bacteria and subsequently the development of dental caries. However, we have not studied its toxic effect in the oral cavity, which is very necessary before clinical application (for example, as an antibacterial ingredient in oral health-care products). Further studies will contribute to understanding the molecular mechanism for treatment and prevention of dental caries.

## Methods

### Resveratrol, bacterial strain, and growth conditions

Resveratrol (Sigma-Aldrich, USA) was dissolved in dimethyl sulfoxide (DMSO, Sigma-Aldrich, USA) to a stock concentration of 80 mg/mL and diluted in medium to the appropriate concentrations for each experiment. The bacterial strain *S. mutans* UA159 was grown in brain-heart infusion broth (BHI; Oxoid) anaerobically (85% N_2_, 10% H_2_ and 5% CO_2_) at 37 °C in this study. The concentration of DMSO used was up to 1% (800 μg/mL in the resveratrol group), BHI broth or saline solution with 1% DMSO but without resveratrol acted as vehicle control.

### Growth curve assay and minimum inhibitory concentration (MIC)

The effect of various concentrations of resveratrol on the growth of *S. mutans* was assessed by growth curve assay. Briefly, aliquots of overnight culture of *S. mutans* were diluted in BHI broth to the final concentration of 1 × 10^7^ CFU/mL. Various concentrations of resveratrol (0, 50, 100, 200, 400, 800 μg/mL) was added into BHI broth and anaerobically inoculated at 37 °C for 24 h. The bacterial growth was measured using spectrophotometer (UV-1750, Shimadzu, Japan) at OD600 nm every 3 h throughout 24 h of incubation. The growth curve assays were repeated three times independently.

### Glycolysis pH drop assay

The effect of resveratrol on *S. mutans* glycolysis pH drop was measured according to earlier methodology [36]. Briefly, S. mutans was harvested at mid-logarithmic phase by centrifugation, washed with a salt solution (50 mM KCl + 1 mM MgCl_2_), and resuspended in the same salt solution containing different concentrations of resveratrol (0, 50, 100, 200, 400 μg/mL), The pH of the mixture was adjusted to 7.2 with 0.1 M KOH solution and glucose was added in the mixture to a final concentration of 1% (w/v). The decrease in pH by glycolytic activity of *S. mutans* UA159 was assessed at 10 min intervals over a period of 120 min. The experiments were repeated for three times independently.

### Lactate dehydrogenase (LDH) assay

*S. mutans* cells were collected at late exponential phase and incubated at 37 °C in Tris-HCl buffer (pH 7.0) containing 0.5 mg/mL of lysozyme for 1 h [25]. The lysate was then sonicated on ice for 2 cycles of 60 s each, and the cell-free supernatant was collected by centrifugation for 10 min at 4 °C. The crude extract was further dialyzed at 4 °C overnight against 10 mM phosphate buffer (pH 6.9). The dialyzed preparation was defined as crude LDH, and its total protein concentration was measured by the Bradford method to normalize the enzyme activity.

For the LDH assay, crude LDH was pretreated with concentrations of resveratrol (0, 50, 100, 200, 400 μg/mL) at room temperature for 30 min. The reaction mixture (200 μl) contained 180 μl of 50 mM phosphate-buffered saline (pH 6.9) with 0.167 mM NADH and 10 mM sodium pyruvate; 10 μl of fructose 1,6-diphos- phate (final concentration of 1 mM); and 10 μl of pretreated LDH.

Results were expressed as enzymatic activity relative to that of the untreated control. The experiments were performed in triplicates independently.

### Acid tolerance assay

The effect of resveratrol on the acid tolerance of *S. mutans* was evaluated by measurement of the viability of bacteria after 120 min of exposure at pH 5.0 [37]. S. mutans was grown in BHI medium until reaching the mid-logarithmic phase. The cells were collected by centrifugation and resuspended (1 × 10^7^ CFU/mL) in TYEG (10% tryptone, 5% yeast extract, 3% K_2_HPO_4_, and 1% glucose medium buffered with 40 mM phosphate-citrate buffer (pH 5.0) containing different concentration of resveratrol(0, 50, 100, 200, 400 μg/mL). After incubation at 37 °C for 2 h, cells were serially diluted and plated on BHI agar plates for viable counts. The experiments were repeated for three times independently.

### F-ATPase activity

*S. mutans* cells was permeabilized by subjecting the cells to 10% toluene (v/v) followed by two freezing and thawing cycles according to the method described by Belli et al [38]. The F-ATPase activity was evaluated in terms of inorganic phosphate release in the following reaction mixture: 75 mM of Tris-maleate buffer (pH 7.0) containing 5 mM ATP, 10 mM of MgCl_2_, permeabilized cells, and different concentrations of resveratrol (0, 50, 100, 200, 400 μg/mL). After 30 min of reaction, the released phosphate was determined using the method of Bencini et al [39]. The experiments were repeated for three times independently.

### Polysaccharide analyses

The extracellular polysaccharide of *S. mutans* was extracted as previously described with minor modifications [40]. *S. mutans* was incubated with different concentration of resveratrol (0, 50, 100, 200, 400 μg/mL) for 24 h at 37 °C. An equivalent reaction mixture without resveratrol was set as control. The reaction mixture was centrifugated at 10000 rpm for 10 min to separate water-soluble polysaccharide (part 1, supernatant) and water-insoluble polysaccharide (part 2, precipitate). All the supernatant (Part 1) was pooled and added three volumes of cold ethanol. After centrifugation at 4 °C, the supernatant was discarded, and the precipitate (water-soluble polysaccharide) was collected and washed by cold 75% ethanol. The water-soluble polysaccharides were measured using the phenol-sulfuric acid method (0.1% glucose was used for the standard curve). The precipitate (part 2) was dried for 3 h in a Speed Vac concentrator, and used for determination of water-insoluble polysaccharides. The water-insoluble polysaccharides were extracted using 1 M NaOH with agitation at room temperature for 2 h. The water-insoluble polysaccharides were also centrifuged, precipitated, washed and quantified as described above. The experiments were repeated for three times independently.

### Crystal violet assay

Crystal violet assays was used to determine the effect of resveratrol on *S. mutans* biofilm formation in a 96-well microtiter plate [15]. Briefly, overnight culture of *S. mutans* was added into BHI broth with different concentrations of resveratrol (0, 50, 100, 200, 400 μg/mL). After incubation at 37 °C for 6 h, 24 h, the supernatants were removed and washed by sterile PBS three times. Biofilm was stained with 0.1% (w/v) crystal violet for 5 min at room temperature. After washed by sterile PBS three times, 200 μl of 95% ethanol was added to each crystal violet-stained well. Plates were shaken for 10 min, and biofilm formation was quantified by measuring optical density at 595 nm. The experiments were repeated for three times independently.

### Biofilm structure

The *S. mutans* biofilms with different concentrations of resveratrol (0, 50, 100, 200, 400 μg/mL) were inoculated on glass slides in 6-well plates at 37 °C to observe its structure by confocal laser scanning microscopy (CLSM). After incubation for 24 h, the supernatants were removed, washed by sterile PBS three times and stained by the LIVE/DEAD BacLight™ Bacterial Viability Kit for 15 min in the dark according to the manufacture recommendation. This Kit contains SYTO 9 which dyed live cells with intact membranes green fluorescent and propidium iodide (PI) which dyed dead cells with damaged cell membrane red fluorescence. Three random fields of each sample were imaged on a Leica SP5 confocal laser scanning microscopy.

### RNA isolation and real time PCR

To analyse the effect of resveratrol on virulence genes (*ldh*, *relA*, *gtfC*, *comDE*) expression, total RNA of *S. mutans* with different concentrations of resveratrol (0, 50, 100, 200, 400 μg/mL) was extracted by TRIzol reagent (Sigma-Aldrich). cDNA conversion of isolated RNA was done by a cDNA synthesis kit (Takara, Dalian, China) according to the manufacturer’s instructions. The real-time PCR was performed in Applied Biosystems 7500 Real-Time PCR System (Applied Biosystems). All primers used are listed in Table 1. The reaction mixture contained SYBR Green PCR Master Mix (Takara), template cDNA and forward and reverse primers. The PCR conditions included an initial denaturation at 95 °C for 10 min, followed by 40 cycles of denaturation at 95 °C for 15 s, annealing at 60 °C for 30 s. Relative mRNA expression were using the ΔΔCt method. Each experiment was performed with three independent RNA samples in triplicate.

**Table 1:** Specific primers of quantitative real-time PCR

Gene^a^	Primer sequence(5′-3′)		Description
	Forward	Reverse	
*16S rRNA*	AGCGTTGTCCGGATTTATTG	CTACGCATTTCACCGCTACA	Normalizing internal standard
*ldh*	GGCGACGCTCTTGATCTTAG	GGTTAGCAGCAACGAGGAAG	Lactate dehydrogenase
*relA*	ACAAAAAGGGTATCGTCCGTACAT	AATCACGCTTGGTATTGCTAATTG	Guanosine tetra (penta)-phosphatesynthetase
*gtfC*	GGTTTAACGTCAAAATTAGCTGTATTAGC	CTCAACCAACCGCCACTGTT	Water soluble and insoluble glucan production
*comDE*	ACAATTCCTTGAGTTCCATCCAAG	TGGTCTGCTGCCTGTTGC	Competence-stimulating peptide

^a^Based on the NCBI *S. mutans* UA159 genome database

### Statistical analysis

Statistical analyses were performed using SPSS Statistics 20.0 (IBM, USA). The results for groups with or without resveratrol were statistically analyzed by one-way analysis of variance (ANOVA) with post hoc test. A *P*-values of < 0.05 were considered statistically significant.

## Data Availability

The datasets used and/or analysed during the current study available from the corresponding author on reasonable request.

## Abbreviations

CLSM: Confocal laser scanning microscopy
CV: Crystal violet
EPS: Extracellular polysaccharide
GTFs: Glucosyltransferases
LDH: Lactate dehydrogenase
MIC: Minimum inhibitory concentration
*S. mutans*: *Streptococcus mutans*
